# PeSTo-Carbs: Geometric Deep Learning for Prediction of Protein–Carbohydrate Binding Interfaces

**DOI:** 10.1021/acs.jctc.3c01145

**Published:** 2024-04-11

**Authors:** Parth Bibekar, Lucien Krapp, Matteo Dal Peraro

**Affiliations:** † Department of Biological Sciences, Indian Institute of Science Education and Research (IISER) Kolkata, Mohanpur 741246, India; ‡ Institute of Bioengineering, School of Life Sciences, Ecole Polytechnique Fédérale de Lausanne (EPFL), Lausanne 1015, Switzerland; § Swiss Institute of Bioinformatics (SIB), Lausanne 1015, Switzerland

## Abstract

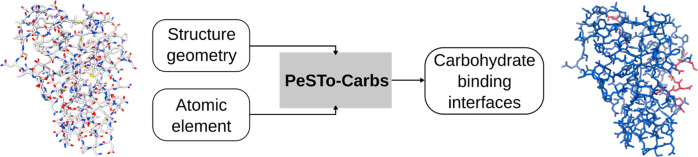

The Protein Structure Transformer (PeSTo), a geometric transformer, has exhibited exceptional performance in predicting protein–protein binding interfaces and distinguishing interfaces with nucleic acids, lipids, small molecules, and ions. In this study, we introduce PeSTo-Carbs, an extension of PeSTo specifically engineered to predict protein–carbohydrate binding interfaces. We evaluate the performance of this approach using independent test sets and compare them with those of previous methods. Furthermore, we highlight the model’s capability to specialize in predicting interfaces involving cyclodextrins, a biologically and pharmaceutically significant class of carbohydrates. Our method consistently achieves remarkable accuracy despite the scarcity of available structural data for cyclodextrins.

## Introduction

Carbohydrates are the primary source of energy for all organisms.^1^ Studying protein–carbohydrate interactions are essential to understanding many fundamental biological processes. Carbohydrates form interaction interfaces with various proteins in metabolic pathways, but studying these interactions through experimental techniques can be challenging due to their weak binding affinities.^2^ Now, with the availability of large data sets containing experimentally solved protein–carbohydrate complexes^3,4^ and the rapid development of machine learning methods to learn from these data, there is a motivation for developing computational methods to study protein–-carbohydrate interactions.

In recent years, researchers have exploited various computational methods to study protein–carbohydrate interactions. These methods have mainly used structure-based, sequence-based, and homology-based approaches to predict protein–carbohydrate binding sites.^5^ Taroni et al. made the first effort in this direction, which predicted the binding sites through structural analysis of protein–carbohydrate complexes in the Protein Data Bank.^106^ Later iterations of structure-based approaches involved various methods like docking,^7^ empirical analysis,^8^ and energy-based analysis.^9^ The latest advancement in structure-based methodologies by Canner et al.^10^ introduced two deep learning models, CAPSIF:Voxel (CAPSIF:V) and CAPSIF:Graph (CAPSIF:G). CAPSIF:V employs a 3D voxelized approach to encode the β-carbon (Cβ) of each residue into a voxel, enabling binary classification to determine the presence of a carbohydrate binding site within each voxel. On the other hand, CAPSIF:G leverages an equivariant graph neural network (EGNN), where the nodes of the graph correspond to the Cβ atoms of individual residues and edges link neighboring residues within a 12 Å radius.

Various sequence-based methods have also been developed, incorporating evolutionary information obtained from the position-specific scoring matrix (PSSM). Since the inception of the first sequence-based method by Malik and Ahmad in 2007,^6^ various methods incorporating modern machine learning algorithms have been developed. Taherzadeh et al. developed a support vector-based prediction model called SPRINT-CBH.^11^ SPRINT-CBH is based on PSSM and additional properties like solvent accessible surface area (SASA), secondary structure (SS), hydrophobicity, protein disordered region, etc. Gattani et al. developed a stacking-based model called StackCBPred^12^ incorporating various features like PSSM, SASA, SS, physiochemical properties, etc. More recently, MTDsite read sequence-based features using LSTM to predict binding sites with DNA, RNA, carbohydrate, and peptide.^13^

With the advent of AlphaFold,^14^ predicted protein structures from sequence information became reliable. As the prediction of the structure from sequence keeps improving, it creates an opportunity to revisit structure-based tools for predicting protein interfaces with other molecules. Various methods for protein interaction prediction combining geometric deep learning^15^ with transformers^16,17^ have been developed recently. One such structure-based deep learning approach is the Protein Structure Transformer (PeSTo), a protein interface prediction method built on a geometric transformer.^18^ PeSTo can predict protein interaction interfaces with other proteins, nucleic acids, ions, ligands, and lipids with high confidence. PeSTo is a geometric transformer that directly acts on protein atoms which are described only using the coordinates and atomic element information with no requirement for physical parameters like mass, charge, and hydrophobicity. Therefore, this approach does not require any preprocessing of the structures, making it straightforward to apply to other tasks. Importantly, since PeSTo was already trained to predict various ligand binding interfaces with proteins including some carbohydrates, we set out to extend and specialize it for carbohydrate binding interface prediction.

Here, we introduce PeSTo-Carbs, an extension of PeSTo trained to predict protein–carbohydrate interacting interfaces. We present two models for PeSTo-Carbs. PeSTo-Carbs General (PS-G) is a versatile model optimized to predict a broad spectrum of protein–carbohydrate interfaces, encompassing various carbohydrate types and their biologically significant derivatives, such as amino sugars, azide sugars, N-linked glycans, etc. PS-G demonstrates impressive performances on a comprehensive test data set of 343 subunits. Additionally, we provide PeSTo-Carbs specialized (PS-S), tailored for more specific predictions by training on nonhomologous protein structures associated with only 21 types of carbohydrate monomers. We tested PS-S on an independent test set of 90 high-resolution subunits from Canner et al.,^10^ demonstrating state-of-the-art performance compared to previously developed structure-based methods. The performance of both our models is consistent and comparable across data sets.

Further, to showcase the flexibility of our method, we also trained the PS-G to differentiate protein–cyclodextrin interfaces specifically alongside protein–carbohydrate complexes. Cyclodextrins have been shown to stabilize proteins in liquid and dry states and inhibit the aggregation of proteins by protecting hydrophobic regions of the peptides in their apolar central cavity.^19^ This makes cyclodextrins important molecules with various applications in pharmaceutics, drug delivery, and chemical industries.^20,21^ Despite the limited data on protein–cyclodextrin complexes in the Protein Data Bank, the method demonstrated a promising performance in predicting protein-cyclodextrin binding interfaces.

## Methods

The Protein Structure Transformer (PeSTo)^18^ architecture is able to accurately predict protein binding interfaces with many types of molecules such as proteins, nucleic acid, ions, small molecules, and lipids. Therefore, it is an ideal choice of approach for the predictions of protein binding interfaces with carbohydrates.

PeSTo takes as input structures represented as an atomic point cloud described by the coordinates and the atomic element. It does not require any parametrization and can be easily applied to any structure. At the core of the PeSTo architecture is the geometric transformer^18^ (***GT***). The geometric transformer operation possesses crucial properties: it is translation-invariant and rotation-equivariant and independent of the order of atoms and interactions. This key operation updates the state of each atom by considering the local geometry and the state of atoms within a predefined neighborhood, defined by a set of nearest neighbors (***nn***). The state of each atom is represented by a scalar state (*q*) and a vector state (***p***), while the geometry is characterized by pairwise distances (***D***) and normalized displacement vectors (***R***). When the number of atoms in a structure is fewer than the number of nearest neighbors, the additional, non-existent interactions are directed to a sink node with both scalar and vector states set to zero. In the PeSTo architecture, each layer (***l***) of geometric transformer process and propagate the scalar, vector, and geometrical information on the structure as described in eq 1.
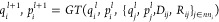
1

The geometric transformer leverages the attention mechanism based on the queries, keys, and values approach^16^ as described in eqs 2 and 3. The queries for the scalar and vectorial tracks (*Q*_*q*_,*Q*_*p*_) are derived from the state of central atom *i* (*q*_*i*_,*p*_*i*_). The keys (***K***) are encoded from the interactions of the central atom *i* with its neighboring atoms *j*, encapsulating the states of the central atom, the neighboring atom, and their spatial relation (). Scalar value vectors (***V***_***q***_) and vector value vectors (***V***_***p***_) are, respectively, extracted from the computed scalar and vector quantities of these interactions. The transformer allows a flexible linear composition of the vector features and states such that the resulting vector state is equivariant to a rotation of the input vector. The attention is done over multiple heads and projected using learned weights for the scalar and vector tracks (*W*_*q*_^*l*^,*W*_*p*_^*l*^).
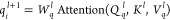
2
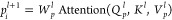
3

### Deep learning architecture

**Figure 1:**
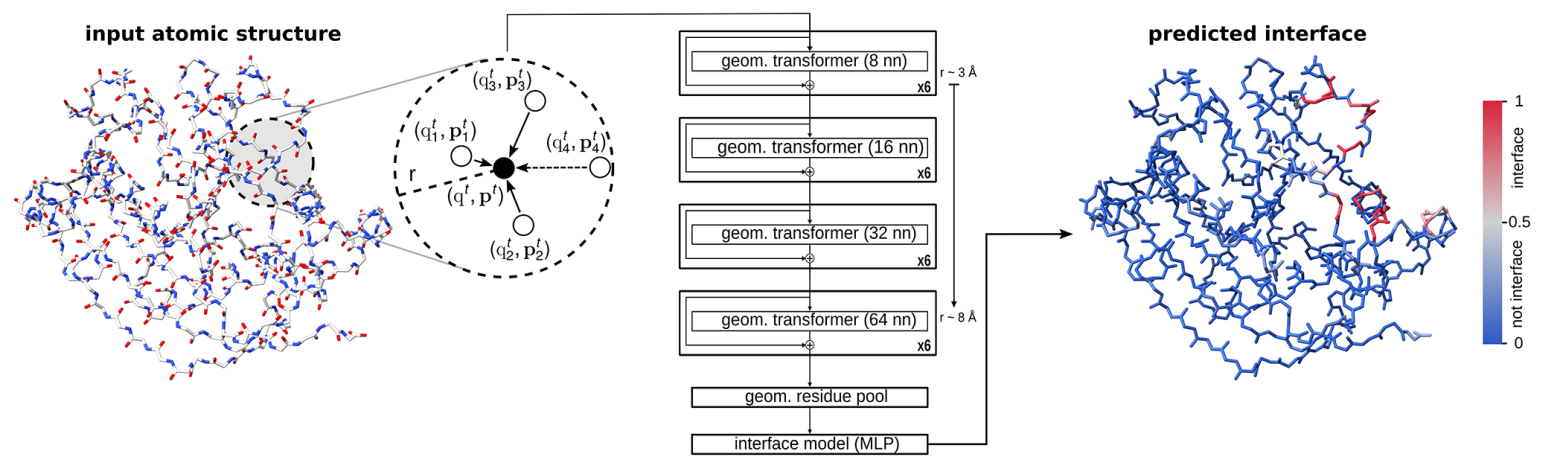
Architecture of PeSTo-Carbs for the prediction of protein–carbohydrate binding interfaces. The model consists of several layers of geometric transformers with a fixed number of nearest neighbors (*nn*) and residual connections. The structure is transformed into a residue representation using a transformer-based geometric pooling. The residue states are then condensed, and a multilayer perceptron (MLP) is employed to generate the final prediction.

We employ a scaled-down version of the PeSTo^18^ architecture for the training of PeSTo-Carbs, aiming to prevent overfitting in the context of limited data. First, we use a three-layer neural network to convert one hot encoding of the atom element into a scalar state size of 32. The initial vector state is initialized to a zero state. Then 24 geometric transformers are applied in series, each having two attention heads, a key size of 3 and a neighborhood of 8 to 64 nearest neighbors as illustrated in Figure 1. Integrating residual connections among geometric transformers allows us to train deeper neural network architectures, while mitigating the vanishing gradient problem. Lastly, a four-headed self-attention within each residue aggregates the atomic-level encodings into a residue description. A three-layer neural network decodes the residue-level scalar and normed vector states to predict the binding interfaces using a sigmoid activation function.

### Data set

PeSTo-Carbs was trained, validated, and tested on protein–carbohydrate complexes collected from the Protein Data Bank (PDB).^3^ We collected all biological assemblies containing carbohydrates and their derivatives (see Supporting Information Table S1) and clustered the subunits using a maximum of 30% sequence identity between clusters. For PS-G, the training, validation, and testing data sets contain 5251, 408, and 343 subunits, respectively. All of the subunits in the test data set have a resolution of less than 3 Å. Similarly, we collected biological assemblies containing protein–cyclodextrin complexes from the PDB. The training, validation, and testing data sets contain 138, 12, and 16 subunits for protein–cyclodextrin complexes, respectively. All performance scores and examples in this work are obtained from the test set.

For PS-S, we selected only the high-resolution (<3 Å), nonhomologous subunits, which gave us 1082 and 98 subunits for our training and validation data sets. To benchmark PS-S, we collected an independent test data set of 90 high resolution subunits derived from Canner et al.;^10^ this data set is represented as TS90. We ensure that there are no structures in TS90 with more than 30% sequence identity with the training data set.

### Structure processing

Every model of the structure is loaded together into one entity. To distinguish them, nonpolymer chemical molecules are given unique chain names for their separate subunits. Water molecules and hydrogen atoms are eliminated from the structures. In the data set, cyclodextrin subunits are labeled distinctively from other glucopyranoses.

### Features and labels

The input structures are described by using the atomic elements, a matrix representing the pairwise distances between atoms, and the pairwise normalized relative displacement between atoms. We restrict the atomic element information to the 30 most common elements and represent them using one-hot encoding. The models work effectively without atom parametrization and can accommodate incomplete molecular structures.

The models aim to predict the residues that are in contact with the carbohydrates. We define an interacting interface between proteins and carbohydrates using a 4 Å cutoff distance: amino acids within 4 Å of a carbohydrate are considered to be in contact. This cutoff aligns with known stacking interactions between proteins and carbohydrates, falling within the 3.3–4 Å range.^22^

### Training

Both models with the same architecture were trained on interfaces between proteins and carbohydrates. We employed binary cross-entropy loss (BCE)^23^ as the objective function for the model. Adaptive Moment Estimation (Adam)^24^ was used as the optimizer with a learning rate of 1 × 10^–4^. Furthermore, we assigned a weight of 0.9 to the positive label in the loss function to account for the class imbalance in our data set.

### Evaluation

PeSTo-Carbs’ performances for both protein–carbohydrate and protein–cyclodextrin interfaces were evaluated on the test data sets as mentioned previously. The performance of our method is assessed by the area under the receiver operating characteristic curve (ROC-AUC), Matthews correlation coefficient (MCC), Dice coefficient, and F1 score, along with various other metrics (see Supporting Information Table S2).

## Results and Discussion

Structures with protein–carbohydrate and protein–cyclodextrin interfaces were extracted from the PDB.^3^ The models are trained to predict the residues that could be part of the interface and flagged upon training as 0 (i.e., the residue is not at a protein–carbohydrate interface) or 1 (i.e., the residue is at an interface). On prediction, the farther the value is from 0.5, the higher the confidence. The two models of PeSTo-Carbs are trained on different sets of carbohydrates (see Methods for details).

PeSTo-Carbs general (PS-G) is evaluated on 359 (with 343 carbohydrates and 16 cyclodextrins) randomly selected chains while ensuring that the sequence identity between the training and test sets was less than 30%. The method achieved a median receiving operating characteristic (ROC) (Supporting Information (SI) Figure S1) area under the curve (AUC) of 0.915 with a balanced accuracy of 0.823 and precision-recall (PR) area under the curve (AUC) of 0.542 (SI Figure S2) for protein–carbohydrate interfaces. To showcase the flexibility of the method, we also trained the model to differentiate protein–cyclodextrin interfaces, specifically alongside protein–carbohydrate complexes. For protein–cyclodextrin interfaces, the model achieved a ROC-AUC of 0.849 (SI Figure S1) with a BACC of 0.782 and a PR-AUC (SI Figure S2) of 0.282, showing promising performance for potential applications even with limited data availability. All of the evaluation metrics for the model are shown in Table 1.

**Table 1:** PeSTo-Carbs (PS-G) Benchmarking Results for Protein–Carbohydrates and Protein–Cyclodextrin Binding Interface Prediction

Evaluation metric	Protein–carbohydrate	Protein–cyclodextrin
TPR	0.713	0.655
TNR	0.934	0.909
PPV	0.365	0.278
ACC	0.922	0.897
BACC	0.823	0.782
NPV	0.984	0.980
MCC	0.475	0.381
F1	0.483	0.390
PR-AUC	0.542	0.282
ROC–AUC	0.915	0.849

**Figure 2:**
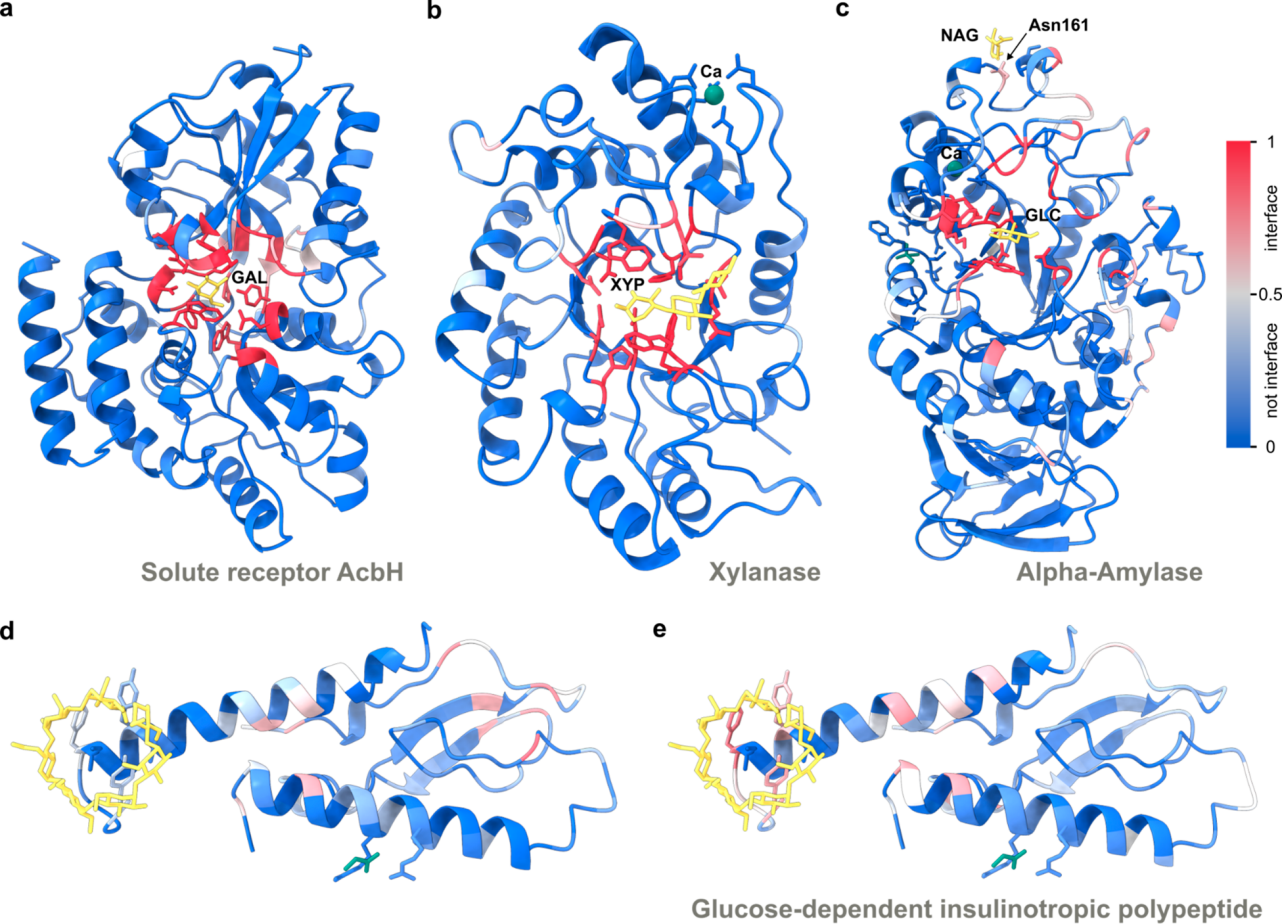
Examples of protein–carbohydrate (a–d) and protein–cyclodextrin (e) interface predictions using PeSTo-Carbs. The model is applied to the protein structure alone. The confidences of the predictions are shown with a gradient of color from blue for non-interfaces to red for interfaces. The carbohydrates (yellow) and other small molecules (green) are subsequently added to assess the quality of the prediction visually. (a) Bacterial solute receptor AcbH complexed with β-d-galactopyranose (GAL) (PDB ID: 3OO6). (b) Xylanase (XynB) complexed with β-d-xylopyranose (XYP) and calcium ion (Ca) (PDB ID: 4PN2), (c) α-Amylase complexed with α-d-glucopyranose (GLC) and calcium ion (Ca). The structure also contains a possible *N*-glycosylation site at Asn161 (PDB ID: 3VM7). Predicted protein–carbohydrate (d) and protein–cyclodextrin (e) for the glucose-dependent insulinotropic polypeptide and receptor in complex with β-cyclodextrin (PDB ID: 2QKH).

To illustrate the performance of the method, Figure 2 shows the prediction of PS-G at the predicted interface with carbohydrates or cyclodextrin for some selected structures. Panels a–c of Figure 2 show the prediction on bacterial solute receptor AcbH complexed with β-d-galactopyranose^25^ (PDB: 3OO6), GH10 endo-*b*-1,4-xylanase (XynB) from *Xanthomonas axonopodis* complexed with xylotriose^26^ (PDB: 4PN2), and the α-amylase from *Malbranchea cinnamomea* complexed with α-d-glucopyranose^27^ (PDB: 3VM7), respectively. It also correctly ignores noncarbohydrate binding sites, such as those with ions, demonstrated in Figure 2b,c. For the α-amylase protein (Figure 2c) the model identifies N161 as a carbohydrate-binding site, aligning with its known status as an *N*-glycosylation site. This suggests the potential of the model for identifying glycosylation sites as well. In the case of the glucose-dependent insulinotropic polypeptide^28^ (PDB: 2QKH), we show that the model can predict specifically cyclodextrin binding; see Figure 2d,e. In this case, the model predicts binding with cyclodextrin but not with other carbohydrates.

We also trained a version of PeSTo-Carbs specialized (PS-S), following the same set of carbohydrates as CAPSIF,^10^ on 21 types of carbohydrate monomers, to create a model for more specific cases. This model might be more fit for the recognition of smaller carbohydrate binding pockets in proteins, for example in the type 1 carbohydrate recognition domain (CRD) in lectins.^29^ The performance of PS-S is evaluated on the TS90 data set which contains 90 high resolution subunits from Canner et al.,^10^ which has less than 30% sequence identity with our train data set. We benchmark the performance of PS-S and compare it with PS-G, CAPSIF:V and CAPSIF:G (Table 2).

**Table 2:** PeSTo-Carbs (PS-G & PS-S) Benchmarking Results on the TS90 Dataset

	PeSTo-Carbs		CAPSIF	
Evaluation metric	PS-G	PS-S	CAPSIF:V	CAPSIF:G
BACC	0.813	**0.823**	0.810	0.765
DICE	0.527	**0.638**	0.608	0.513
MCC	0.509	**0.624**	0.622	0.549
F1	0.527	**0.638**	0.623	0.566
PR-AUC	0.619	0.600	**0.623**	0.518
ROC–AUC	0.918	**0.930**	0.810	0.919

PS-S demonstrated superior overall performance with a higher balanced accuracy (BACC), MCC, Dice score, F1 score, and ROC-AUC values. These metrics collectively underscore its excellence in classification accuracy, sensitivity, and specificity and its ability to discriminate between positive and negative instances of protein–carbohydrate binding. However, it is noteworthy that PS-S exhibited a slightly lower PR-AUC than CAPSIF:V, indicating a potential trade-off with precision in situations where minimizing false positives is paramount.

We also evaluated the performance of PS-S on the test data set of sequence-based methods SPRINT-CBH^11^ and StackCBPred.^12^ On this data set of 88 nonhomologous chains, which were excluded from PeSTo-Carbs’s train data set at a 30% sequence identity, the method achieved an MCC of 0.556 and a ROC-AUC of 0.905. Despite the absence of trained models of these methods for a thorough benchmark, it is worth noting that PS-S consistently demonstrates superior performance when compared to the reported results of both SPRINT-CBH (MCC, 0.257; ROC-AUC, 0.744) and StackCBPred (MCC, 0.139; ROC-AUC, 0.752).

**Figure 3:**
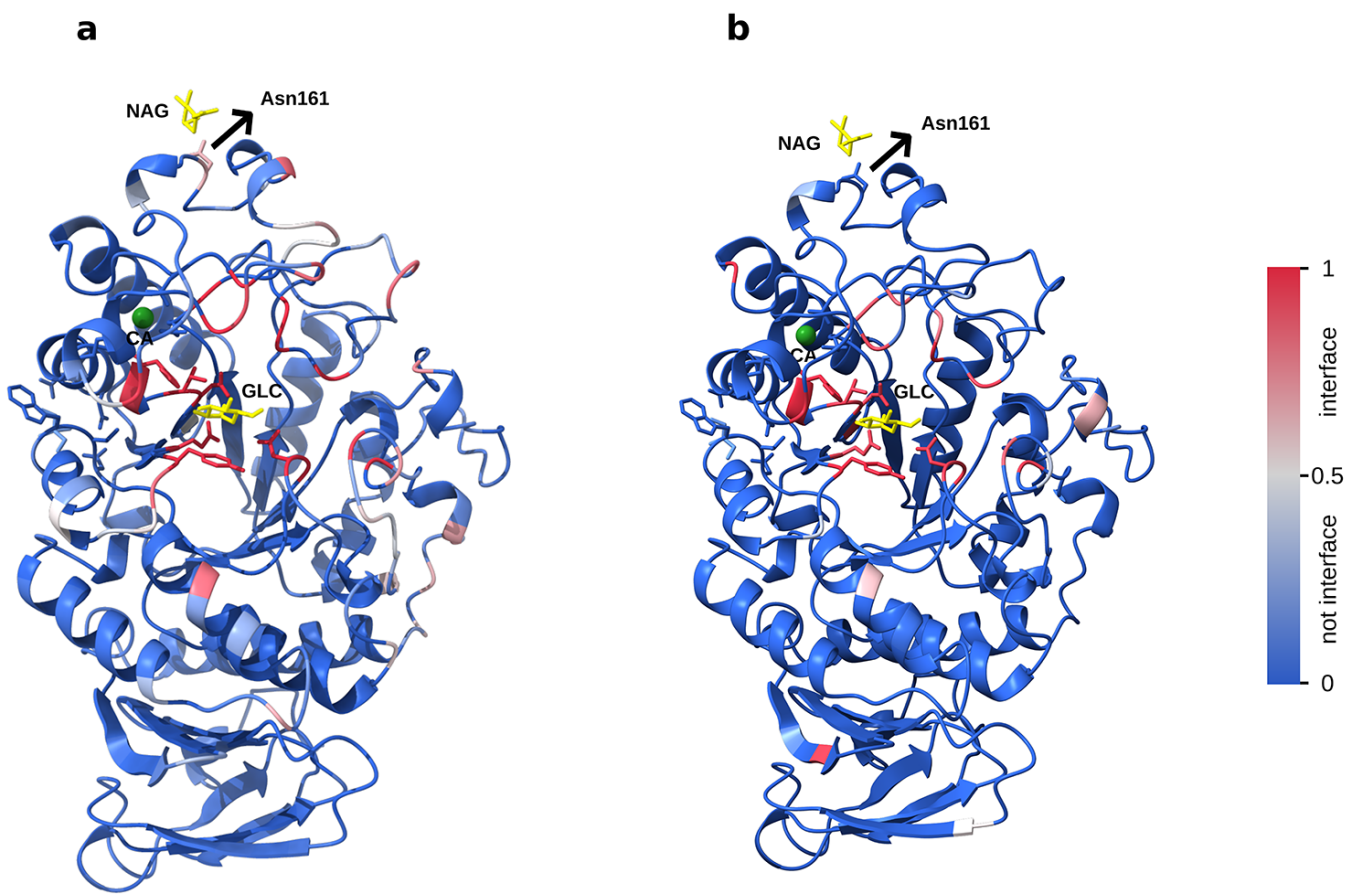
α-Amylase complexed with α-d-glucopyranose (GLC) and calcium ion (Ca). (PDB: 3VM7). Predicted binding interfaces from (a) PS-G and (b) PS-S.

Finally, we compare the differential predictions of PS-G and PS-S. As illustrated in Figure 3a,b, we examined their predictions on the α-amylase^27^ (PDB: 3VM7) by PS-G and PS-S, respectively. Both models correctly ignore the binding interface with the calcium ion. PS-G predicts an interface at N161 which binds to *N*-acetyl-d-glucosamine (NAG). This interface is correctly ignored by PS-S, as NAG interfaces were not a part of the training data for it. Furthermore, PS-S showcased a smaller predicted binding pocket for α-d-glucopyranose (GLC), resulting in fewer false positives compared to the larger interface predicted by PS-G. This suggests that using both models in tandem may improve prediction accuracy for protein–carbohydrate interactions.

We also highlight the robustness of our method in effectively handling the inherent conformational variability in protein structures. Hevein, a protein with a notable 32-amino acid segment serving as a carbohydrate binding domain^30^ was studied through molecular dynamics simulations by Solanke et al.^31^ This investigation focused on the binding mechanism between the hevein-32 domain and *N*-acetylglucosamine monosaccharide (GlcNAc), revealing insights into the protein’s structural dynamics and its interactions with carbohydrates. The Supporting Information (Figure S3) presents a comprehensive analysis of the hevein-32 domain. We show that the PS-S model successfully predicts carbohydrate binding interfaces for hevein-32, including instances with different interacting and non-interacting conformations of protein with carbohydrates. This emphasizes the method’s ability in accommodating conformational variability within protein structures.

## Conclusion

Despite the persistent challenge of accurately predicting carbohydrate-binding residues, PeSTo-Carbs has achieved noteworthy results, demonstrating its potential as a valuable tool in this field. By leveraging advanced deep learning techniques and incorporating relevant features, PeSTo-Carbs has demonstrated a high level of accuracy in predicting both carbohydrate binding and nonbinding residues, resulting in a balanced and comprehensive predictor of carbohydrate binding sites. These findings suggest that PeSTo-Carbs has the potential to contribute significantly to ongoing efforts to understand carbohydrate–protein interactions and their biological significance better.

PeSTo-Carbs was trained to predict binding interfaces between proteins, carbohydrates, and their derivatives. This model achieved a high MCC of 0.475 with an ROC-AUC of 0.915 on our extensive test data set of 343 subunits. Due to the very flexible nature of parameter-free transformers, we could apply the model to also specialize on a specific target—cyclodextrins with very limited data. The method performed moderately well on cyclodextrins (MCC, 0.381; ROC-AUC, 0.849), which shows that the method can be easily adapted for specific applications with limited data. We also trained PeSTo-Carbs specialized in a more specific set of protein–carbohydrate interfaces with only 21 carbohydrate monomers. This model is more fit to predict smaller binding pockets with higher sensitivity while outperforming the previously developed structure-based as well as sequence-based methods.

The pretrained PeSTo-Carbs models on carbohydrates and cyclodextrins and all of the data to reproduce the results are available to the community at https://github.com/LBM-EPFL/PeSTo-Carbs (accessed April 2024). PeSTo-Carbs (PS-G and PS-S) along with PeSTo is available at the web server: https://pesto.epfl.ch/(accessed April 2024) to make protein binding interface predictions with proteins, DNA/RNA, ligands, ions, lipids and now carbohydrates and cyclodextrins.

While PeSTo-Carbs has achieved promising results, there is still room for improvement in accurately predicting carbohydrate-binding residues. Ongoing research efforts continue to explore new strategies and methodologies for enhancing the performance of such predictors. Nonetheless, PeSTo-Carbs’s performance represents a significant advancement in this field and provides a strong foundation for further investigation and development of more accurate carbohydrate-binding residue predictors. We hope that the development of this method will help in searching for protein–carbohydrate and protein–cyclodextrin binding interfaces from protein structures and can be utilized to develop target drugs. We also hope that the specialization of PeSTo-Carbs on cyclodextrins motivates further domain-specific applications with limited data.

## Data Availability

Code to reproduce our data sets and results is available at https://github.com/LBM-EPFL/PeSTo-Carbs (accessed April 2024).

## Supporting Information Available

List of carbohydrates; definitions of all evaluation metrics used and additional analyses (PDF)
