# Supplementary material for: PeSTo-Carbs: Geometric Deep Learning for Prediction of Protein–Carbohydrate Binding Interfaces
Source: J Chem Theory Comput. 2024 Apr 11;20(8):2985–91. doi: 10.1021/acs.jctc.3c01145 (PMC11044267; doi:10.1021/acs.jctc.3c01145)
Supplement: Supplementary file 1 — ct3c01145_si_001.pdf [file ct3c01145_si_001.pdf]

Supporting Information

PeSTo-Carbs: Geometric Deep Learning for Prediction of Protein-Carbohydrate Binding Interfaces

Parth Bibekar,[†,‡] Lucien Krapp,[‡,¶] and Matteo Dal Peraro[*,‡,¶]

†*Department of Biological Sciences, Indian Institute of Science Education and Research (IISER) Kolkata, Mohanpur 741246, India*

‡*Institute of Bioengineering, School of Life Sciences, Ecole Polytechnique Fédérale de Lausanne, EPFL, Lausanne 1015, Switzerland*

¶*Swiss Institute of Bioinformatics (SIB), Lausanne 1015, Switzerland*

* To whom correspondence should be addressed: M.D.P., email: matteo.dalperaro@eplf.ch

Dataset

PeSTo-Carbs General (PS-G) was trained on protein carbohydrate complexes with the following 35 molecules:
N-acetyl-beta-D-glucosamine (NAG), beta-D-glucopyranose (BGC), alpha-D-glucopyranose (GLC), alpha-D-mannopyranose (MAN), beta-D-galactopyranose (GAL), alpha-L-fucopyranose (FUC), beta-D-mannopyranose (BMA), N-acetyl-alpha-D-mannosamine (BM3), nonyl beta-D-glucopyranoside (BNG), beta-D-xylopyranose (XYP), uridine-diphosphate-n-acetylglucosamine (UD1), N-acetyl-alpha-D-galactosamine (A2G), undecyl-maltoside (UMQ), N-acetyl-beta-D-galactosamine (NGA), galactose-uridine-5-diphosphate (GDU), 6-O-phosphono-beta-D-glucopyranose (BG6), N-acetyl-beta-neuraminic acid (SLB), N-acetyl-alpha-neuraminic acid (SIA), fructose -6-phosphate (F6R), alpha-D-galactopyranose (X6X), 1,6-di-O-phosphono-D-fructose (P6F), 2-amino-2-deoxy-alpha-D-glucopyranose (PA1), 2-amino-2-deoxy-beta-D-galactopyranose (1GN), alpha-L-rhamnopyranose (RAM), alpha-D-Abequopyranose (ABE), alpha-D-glucopyranuronic acid (GCU), 2-acetamido-2-deoxy-4-O-sulfo-beta-D-galactopyranose (ASG), alpha-L-gulopyranuronic acid (LGU), alpha-L-arabinofuranose (AHR), beta-D-galactofuranuronic acid (GTK), beta-D-glucopyranuronic acid (BDP), beta-L-fructofuranose (LFR) and cyclodextrins.

PeSTo-Carbs Specialized (PS-S) included the following 21 monomers from above: GLC, BGC, FUC, GAL, ASG, NGA, SIA, AHR, XYP, MAN, RIB, ADA, GTK, BDP, GCU, LGU, RAM, ABE, BM3, FRU, LFR.

Table S1: Carbohydrates in the dataset

Molecule name	Molecule ID	PDB Count
2-acetamido-2-deoxy-beta-D-glucopyranose	NAG	844
beta-D-glucopyranose	BGC	347
alpha-D-glucopyranose	GLC	294
alpha-D-mannopyranose	MAN	219
beta-D-galactopyranose	GAL	185
alpha-L-fucopyranose	FUC	106
beta-D-mannopyranose	BMA	106
nonyl beta-D-glucopyranoside	BNG	102
beta-D-xylopyranose	XYP	92
uridine-diphosphate-n-acetylglucosamine	UD1	90
N-acetyl-alpha-neuraminic acid	SIA	51
2-acetamido-2-deoxy-alpha-D-galactopyranose	A2G	45
beta-cyclodextrin	-	45
undecyl-maltoside	UMQ	44
2-acetamido-2-deoxy-beta-D-galactopyranose	NGA	42
galactose-uridine-5-diphosphate	GDU	39
6-O-phosphono-beta-D-glucopyranose	BG6	38
N-acetyl-beta-neuraminic acid	SLB	33
beta-D-glucopyranuronic acid	BDP	25
alpha-cyclodextrin	-	21
fructose -6-phosphate	F6R	18
alpha-D-ribofuranose	RIB	16
alpha-L-arabinofuranose	AHR	15
alpha-D-glucopyranuronic acid	GCU	11
2-acetamido-2-deoxy-alpha-D-mannopyranose	BM3	10
gamma-cyclodextrin	-	8
alpha-L-rhamnopyranose	RAM	7
alpha-D-galactopyranuronic acid	ADA	7
2-amino-2-deoxy-alpha-D-galactopyranose	X6X	4
2-acetamido-2-deoxy-4-O-sulfo-beta-D-galactopyranose	ASG	4
1,6-di-O-phosphono-D-fructose	P6F	4
beta-L-fructofuranose	LFR	3
2-amino-2-deoxy-alpha-D-glucopyranose	PA1	2
2-amino-2-deoxy-beta-D-galactopyranose	1GN	1
beta-D-galactofuranuronic acid	GTK	1
alpha-D-Abequopyranose	ABE	1

Table S2: Evaluation metrics

Evaluation metric	Definition
True positive rate (TPR)	$\frac{TP}{TP+FN}$
True Negative Rate (TNR)	$\frac{TN}{TN+FP}$
Positive Predictive Value (PPV)	$\frac{TP}{TP+FP}$
Accuracy (ACC)	$\frac{TP+TN}{TP+TN+FP+FN}$
Balanced Accuracy (BACC)	$\frac{1}{2}\left(\frac{TP}{TP+FN}+\frac{TN}{TN+FP}\right)$
Negative Predictive Value (NPV)	$\frac{TN}{TN+FN}$
Matthews Correlation Coefficient (MCC)	$\frac{TP\cdot TN-FP\cdot FN}{\sqrt{(TP+FP)(TP+FN)(TN+FP)(TN+FN)}}$
F1 Score	$\frac{2\cdot(PPV\cdot TPR)}{PPV+TPR}$
DICE Score	$\frac{2\cdot TP}{2\cdot TP+FP+FN}$

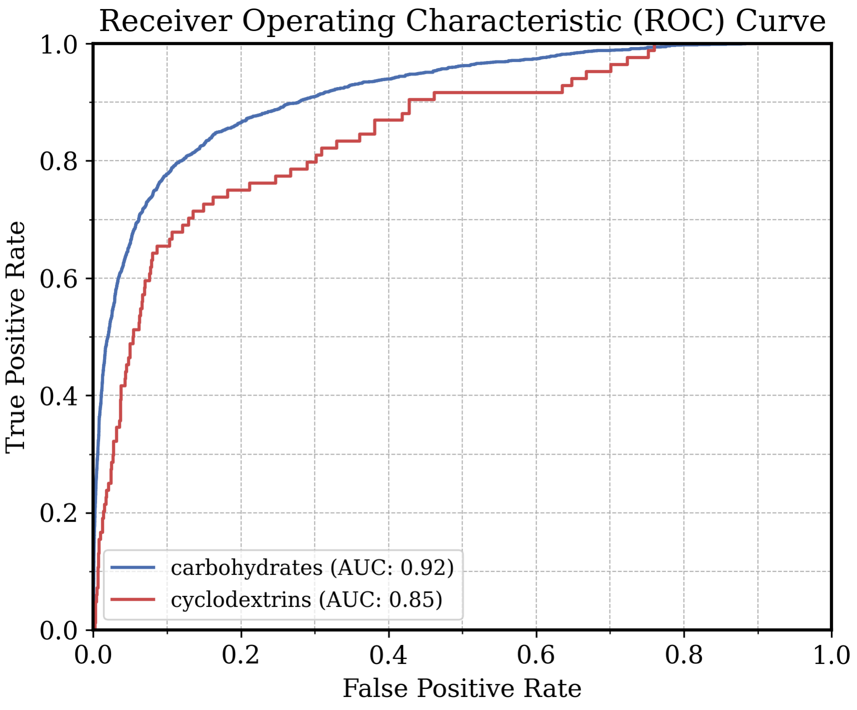

Figure S1: Receiving Operating Characteristic Curve for the predictions of protein-carbohydrate and protein-cyclodextrin interfaces with PS-G on the benchmark dataset.

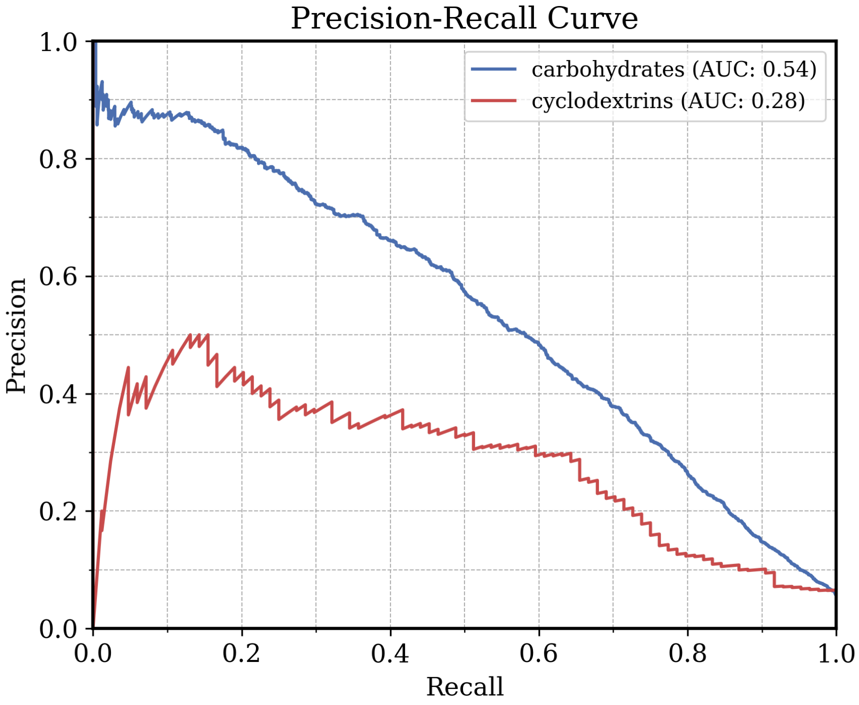

Figure S2: Precision-Recall Curve for the predictions of protein-carbohydrate and protein-cyclodextrin interfaces with PS-G on the benchmark dataset.

Carbohydrate-protein complex formation in Hevein-32 domain

A defined 32-amino acid segment within the hevein protein has been identified as a carbohydrate binding domain[1]. Solanke et al. conducted a 2 μs molecular dynamics simulation to elucidate the binding mechanism between this truncated protein, hevein-32, and N-acetylglucosamine monosaccharide (GlcNAc)[2]. Within the simulation, hevein-32 transitions from an initial unbound state to a specifically binding state with GlcNAc at approximately 720 ns. Utilising this simulation trajectory, we applied the PS-S model to analyse protein conformations at 20 ns intervals, enabling predictions of the carbohydrate binding interface. **Figure S3a** illustrates the Root Mean Square Deviation (RMSD) of the hevein-32 domain throughout the simulation. **Figure S3b** attests that the model accurately identifies the absence of any binding interface in the unbound state. In **Figure S3c**, the model correctly predicts the binding interfaces for Tyr30 and Glu29 residues in the bound state, however it is noteworthy that the prediction for Trp23 falls within the low-confidence range. This example underscores the robustness of our method in handling the conformational variability inherent in protein structures.

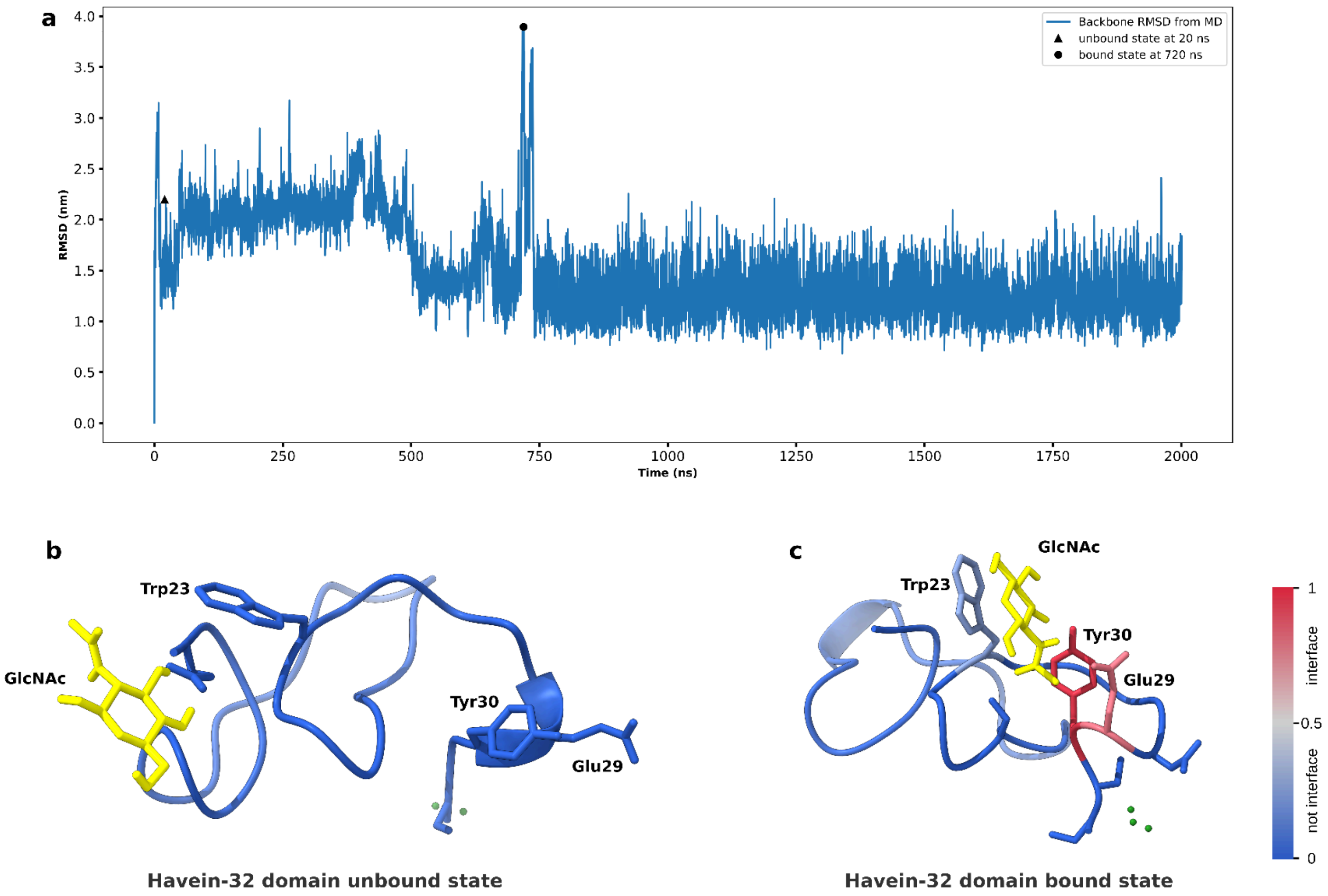

Figure S3: (a) Backbone RMSD of the hevein-32 domain over the course of a 2 µs molecular dynamics simulation. Prediction of PS-S on the unbounded state (b) at 20 ns and the bounded state (c) at 720 ns. The model is applied to the protein structure alone. The confidence of the predictions is shown with a gradient of color from blue for non-interfaces to red for interfaces. The N-acetylglucosamine monosaccharide (in yellow) is subsequently added to assess the quality of the prediction visually.

References

1. Aboitiz, N.; Vila-Perelló, M.; Groves, P.; Asensio, J. L.; Andreu, D.; Cañada, F. J.; Jiménez-Barbero, J. NMR and Modeling Studies of Protein–Carbohydrate Interactions: Synthesis, Three-Dimensional Structure, and Recognition Properties of a Minimum Hevein Domain with Binding Affinity for Chitooligosaccharides. ChemBioChem, 2004, 5, 1245–1255. https://doi.org/10.1002/cbic.200400025.
2. Solanke, C. O.; Trapl, D.; Šućur, Z.; Mareška, V.; Tvaroška, I.; Spiwok, V. Atomistic Simulation of Carbohydrate-Protein Complex Formation: Hevein-32 Domain. Scientific Reports, 2019, 9. https://doi.org/10.1038/s41598-019-53815-w.
